# Nuclear localization of beta-catenin involved in precancerous change in oral leukoplakia

**DOI:** 10.1186/1476-4598-6-62

**Published:** 2007-10-09

**Authors:** Kosei Ishida, Satoshi Ito, Naoyuki Wada, Hiroyo Deguchi, Tsuyoshi Hata, Masaru Hosoda, Tsutomu Nohno

**Affiliations:** 1 Department of Oral Surgery, Kawasaki Medical School, Kurashiki 701-0192, Japan; 2 Department of Molecular and Developmental Biology, Kawasaki Medical School, Kurashiki 701-0192, Japan

## Abstract

**Background:**

Oral leukoplakia is a precancerous change developed in the oral mucosa, and the mechanism that oral leukoplakia becomes malignant through atypical epithelium is not known. Here we compared the β-catenin expression detected by immunohistochemical staining in the normal oral epithelium and in the oral leukoplakia with or without dysplasia.

**Results:**

The normal oral epithelium showed β-catenin expression only in the cell membrane, but not in the nuclei. In the oral leukoplakia without dysplasia, 7 out of 17 samples (41%) showed β-catenin expression in the cell membrane, and 5 samples (29%) showed expression in the nuclei. In the oral leukoplakia with dysplasia, nuclear expression of β-catenin was shown in 11 out of 12 samples (92%). Incidence of nuclear β-catenin expression was significantly different between dysplasia and normal oral epithelium (P < 0.01), and also between oral leukoplakia with dysplasia and those without dysplasia (P < 0.01). Wnt3 expression was detected in the epithelial cell membrane or cytoplasm in oral leukoplakia where nuclear expression of β-catenin was evident, but not in epithelial cells without nuclear expression of β-catenin.

**Conclusion:**

The components of canonical Wnt pathway, such as Wnt3, β-catenin, and cyclin D1, were detected, implying that this pathway is potentially involved in the progression of dysplasia in oral leukoplakia.

## Background

Oral leukoplakia is known as a precancerous change of squamous cell carcinoma developed in oral mucosa [1-3]. Leukoplakia is a term expressing clinical disease state, and it occurs in every intra-oral locus and shows various observations. Because a clinician is difficult to be settled with precancerous lesion in these, we require histopathology examination. Leukoplakia diagnosed as epithelial dysplasia histopathology in leukoplakia becomes precancerous. It is said that it may become malignant transformation in progression of the severity in epithelial dysplasia [4]. Although much works have been done on the leukoplakia, the mechanism that oral mucosa epithelium constituting leukoplakia becomes malignant through atypical epithelium is not known.

There are many reports on β-catenin accumulation into a nucleus of a cancer cell in the epithelial malignant tumor, including colorectal cancer [5-8], and transcription activation of a target gene by β-catenin/TCF is known to participate in malignant transformation [9,10]. Transcription activation by β-catenin is triggered by binding of Wnt family to the cell membrane receptors, called Frizzled and LRP5/6, which transduces Wnt signal inside the cell through inactivation of GSK-3. APC and Axin constitute a complex to phosphorylate β-catenin with GSK-3. In the presence of Wnt, the receptor complex transduces negative signals to APC/Axin/GSK-3 complex through Dishevelled, and thus GSK-3 becomes unable to phosphorylate cellular β-catenin. Accumulated β-catenin in cytoplasm is now translocated into a nucleus, forms a transcriptional activation complex with TCF/LEF1 [10-13], and activates various target genes such as c-myc and cyclin D1 [14-17]. In the absence of Wnt, β-catenin in cytoplasm is immediately phosphorylated by GSK-3/Axin/APC complex and receives ubiquitination, eventually leading to degradation in proteasome, and thus disappeared in cytoplasm immediately [9,10].

Because Wnt family is known to participate in epithelial cell proliferation, we examined in this study immunohistochemical localization of β-catenin, with attention to transcription activation of a target gene by β-catenin, and to evaluate nuclear accumulation β-catenin in relevance with atypical epithelium in a surface layer of oral leukoplakia. Nuclear localization of β-catenin is correlated with cyclin D1 expression in oral leukoplakia, and also Wnt3 expression in neighboring cells, known as a typical member of the Wnt family that activates β-catenin-mediated signaling [18,19], suggesting the involvement of Wnt/β-catenin signaling in the progression of dysplasia.

## Results

### Immunohistochemical localization of β-catenin

The expression patterns of β-catenin in oral mucosa are summarized in Table 1. In the normal oral epithelium, nuclear localization of β-catenin was not detected in all 6 samples examined; 5 out of 6 samples showed expression signals only in the cell membrane, and one sample showed cytoplasmic expression in addition to signals in the cell membrane. In the oral leukoplakia without dysplasia, 7 out of 17 samples (41%) showed expression signals in the cell membrane, and 5 samples (29%) showed expression signals in the nuclei. Oral leukoplakia with dysplasia did not show expression signals in the cell membrane, and nuclear expression was shown in 11 out of 12 samples (92%).

**Table 1:** Expression patterns of β-catenin in oral mucosa

Tissue	Total number	Cytoplasm	Nucleus	Membrane
Normal oral epithelium	6	1	0	5
Oral leukoplakia				
Without dysplasia	17	5	5	7
With dysplasia	12	1	11*	0
Oral squamous cell carcinoma	15	5	10	0

*P < 0.01 between normal oral epithelium and oral leukoplakia without dysplasia by Fisher's exact test.

**Figure 1:**
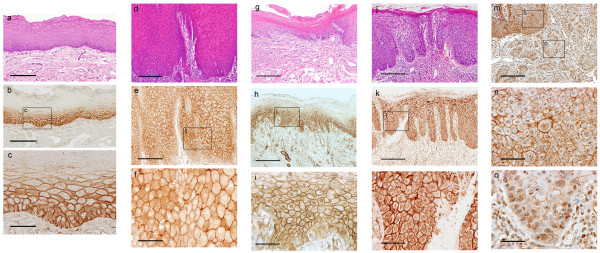
Immunohistochemical localization of β-catenin in normal oral epithelium (a-c), oral leukoplakia without dysplasia (d-f), oral leukoplakia with mild dysplasia (g-i), oral leukoplakia with severe dysplasia (j-l), and oral squamous cell carcinoma (m-o). (a, d, g, j) Hematoxylin and eosin staining. (b, c, e, f, h, i, k, l, m-o) β-Catenin staining. (b, c) Signals were detected in the cell membrane of the basal and spinous layer, but not in the cytoplasm and nuclei. (e, f) Signals were detected in the cell membranes and cytoplasm. (h, i) Signals were detected in the cell membranes and nuclei. (j) The area for dysplasia is characterized by an increased nuclear-cytoplasmic ratio, an increased number of mitotic figures, including abnormal mitoses, nuclear hyperchromatism. (k, l) Signals were detected in the cell membranes and nuclei. (m-o) Signals were detected in the nucleus of the epithelial dysplastic cells (n) and carcinoma cells (o) in OSCC, but cell membranous expression was weak or absent. Scale bars: (a, b, d, e, g, h, j, k, m) 200 μm; (c, f, i, l, n, o) 50 μm.

Localization of β-catenin in the epithelial cell membranes was observed in normal oral epithelium and oral leukoplakia, whereas expression in OSCC was low or totally absent in the cell membrane (Fig. 1). The expression of β-catenin in normal oral epithelium was observed on the cell membrane, but not within the nuclei of basal and spinous layer (Fig. 1b, 1c). In oral leukoplakia without dysplasia, the expression of β-catenin was observed on the cell membrane or both cell membrane and cytoplasm in 24% of basal and spinous layer cells (Fig. 1e, 1f). In oral leukoplakia with mild dysplasia, the expression of β-catenin was observed in the nuclei at about 30% (Fig. 1h, 1i). Oral leukoplakia with dysplasia that was especially characterized by an increased nuclear-cytoplasmic ratio, an increased number of mitotic figures, including abnormal mitoses, nuclear hyperchromatism showed nuclear expression pattern (Fig. 1k, 1l), and the expression of β-catenin in nuclei was shown in more than 80% of epithelial cells. The mean percentage of nuclear staining with β-catenin in oral leukoplakia is presented in Table 2. The nuclear expression of β-catenin in epithelial dysplasia increased depending on the grade of dysplasia, and there were significantly different between β-catenin staining without dysplasia and that with mild dysplasia (P < 0.01), and also between β-catenin staining with mild dysplasia and that with severe dysplasia (P < 0.05). The tumor parenchymal cells of OSCC (Fig. 1m–o) also showed nuclear expression pattern in 10 out of 15 specimens (67%). Incidence of nuclear localization of β-catenin was significantly different between dysplasia and normal oral epithelium (P < 0.01), and also between oral leukoplakia with dysplasia and those without dysplasia (P < 0.01).

**Table 2:** Mean percentage of nuclear β-catenin staining in oral leukoplakia with and without dysplasia

Pathological diagnosis	Total number	Positive ratio (mean value)
No dysplasia	17	6.7
Mild dysplasia	9	26.9*
Severe dysplasia	3	59.7**

*P < 0.01 between no dysplasia samples and mild dysplasia samples by Student's *t*-test.

**P < 0.05 between mild dysplasia samples and severe dysplasia samples by Student's *t*-test.

### Relationship between localization of β-catenin and Wnt3 expression

**Figure 2:**
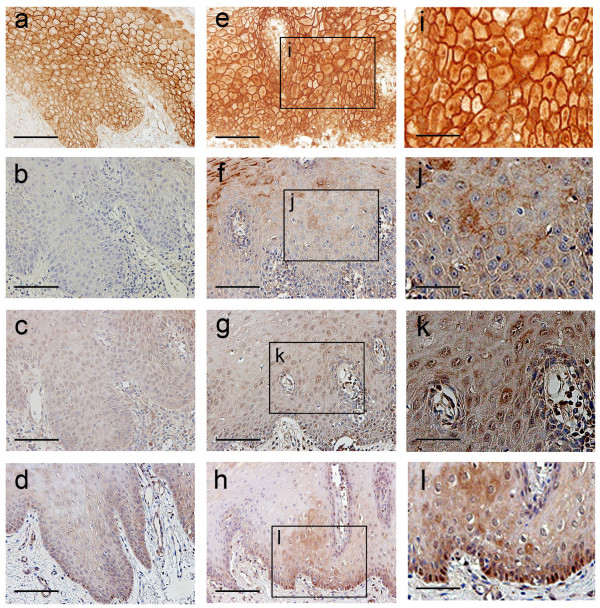
Comparison of immunohistochemical staining for β-catenin, Wnt3, cyclin D1, and c-myc in oral leukoplakia. (a-d) Serial sections of oral leukoplakia without dysplasia. (a) Nuclear expression of β-catenin was not observed. (b) Wnt3 expression was not observed. (c) Cyclin D1 shows weak expression. (d) Nuclear expression of c-myc is observed in the basal layer cells. (e-h) Serial sections of oral leukoplakia with dysplasia. (e, i) β-Catenin is expressed in the nuclei. (f, j) Wnt3 expression is observed on the epithelial cell membrane and in the cytoplasm. (g, k) Cyclin D1 is expressed in several epithelial cells. (h, l) c-Myc shows similar expression pattern as oral leukoplakia without dysplasia. Scale bars: (a-h) 100 μm; (i-l) 50 μm.

We examined the expression pattern of Wnt3 in oral leukoplakia. Wnt3 was not expressed in the normal oral epithelium (data not shown). Figure 2 shows comparison of immunohistochemical staining for β-catenin, Wnt3, cyclin D1, and c-myc in oral leukoplakia without dysplasia (a-d) and those with dysplasia (e-l). Wnt3 was expressed on the epithelial cell membrane or cytoplasm where nuclear expression of β-catenin is evident, in contrast to those without nuclear expression of β-catenin (Fig. 2b, 2f, 2j). In the samples with nuclear expression of β-catenin, Wnt3 expression was shown in 13 out of 16 samples (81%), and there is significant positive correlation between the nuclear expression of β-catenin and Wnt3 staining (P < 0.01) (Table 3).

**Table 3:** Relationship between nuclear expression of β-catenin and Wnt3 expression in oral leukoplakia

β-Catenin	Total number	Wnt3	
		
		-	+
Nuclear expression negative	13	10	3 (23.1%)
Nuclear expression positive	16	3	13 (81.3%)*

*P < 0.01 between nuclear expression negative and positive samples by Mann-Whitney *U*-test.

We also examined other members of Wnt family, including Wnt1, Wnt5a, and Wnt7a, for immunohistochemical staining. No signal was detectable with antibodies against Wnt1 and Wnt7a in normal and leukoplakia epithelia, whereas Wnt5a signal was ubiquitously detectable in normal oral epithelium, leukoplakia, and in OSCC (data not shown). Thus, these Wnt members are unlikely to be involved in nuclear localization of β-catenin because of the absence of correlation.

### Relationship with cyclin D1 and c-myc expression

**Figure 3:**
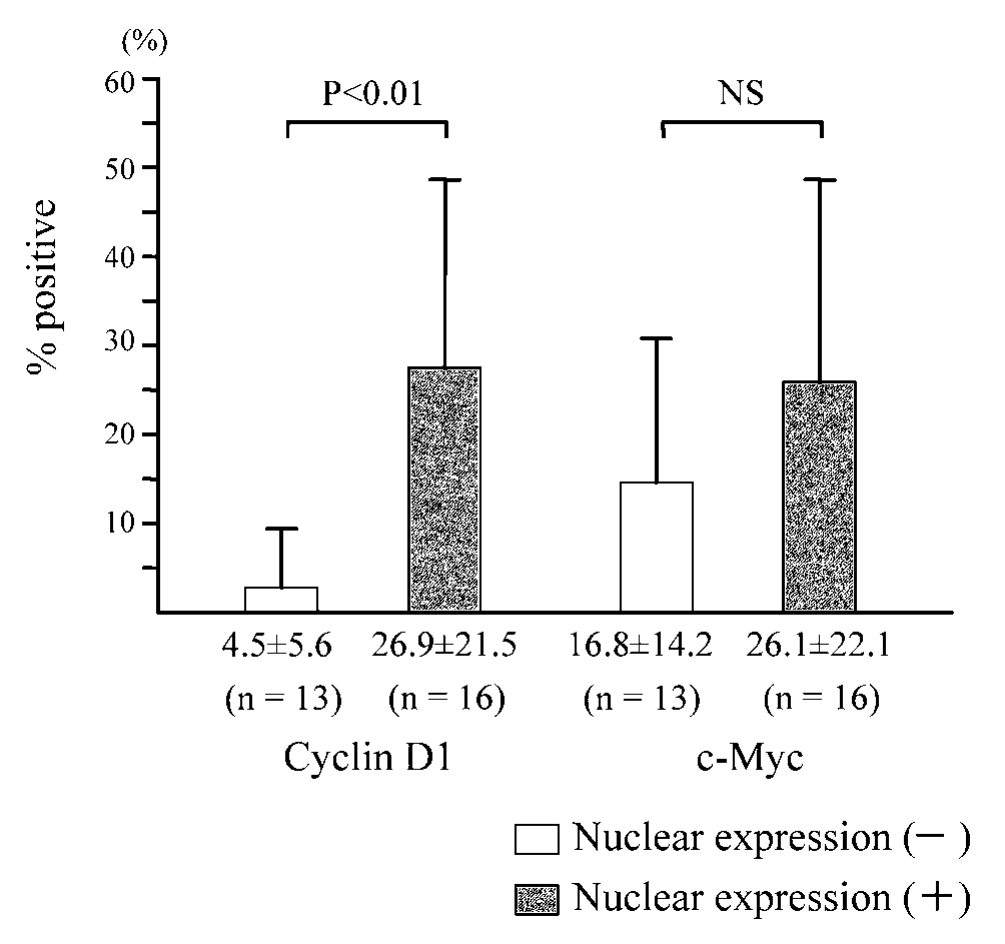
Relationship between subcellular localization of β-catenin and expression patterns of cyclin D1 and c-myc in oral leukoplakia. Shown by means with standard deviations.

Cyclin D1 was intensely expressed in the oral leukoplakia where nuclear expression of β-catenin is evident, in contrast to those without nuclear expression of β-catenin (Fig. 2). Cyclin D1 overexpression was more evident in the oral leukoplakia with dysplasia than that without dysplasia (Fig. 2c, 2g, 2k), and percentage of the cell number with positive nuclear staining is significantly different in these specimens (Fig. 3). On the other hand, the c-myc expression in nuclei was not significantly different between these two specimens (Fig. 2d, 2h, 2l), suggesting independent activation of c-myc.

## Discussion

β-Catenin has been identified to be a constituent of cell adhesion apparatus bound to cadherin family [20], and plays an important role for cellular movement and adhesion, as well as a signaling factor involved in canonical Wnt pathway [9]. Nuclear localization of β-catenin is known to associate with malignant transformation of colorectal cancer and other tumor [5-8]. Because cytoplasmic accumulation and subsequent nuclear translocation of β-catenin is known to be resulted from activation of canonical Wnt signaling pathways [10], subcellular localization of β-catenin is a useful marker to detect cellular conditions to proliferate actively. We compared here immunohistochemical localization of β-catenin in the normal oral epithelia, oral leukoplakia, and OSCC, aimed at elucidating relationship between proliferative activity and subcellular localization of the products.

In contrast to membranous and cytoplasmic expression, nuclear expression of β-catenin is implicated in tumor progression, but the significance of β-catenin localization in the oral epithelial dysplasia and OSCC has not yet been examined in details. The nuclear expression of β-catenin increased during progression of the severity in oral leukoplakia. Furthermore, because localization of β-catenin in normal oral epithelium and dysplasia showed significant difference, suggesting nuclear expression of β-catenin as aberrant condition. In addition, nuclear expression of β-catenin was also observed in 10 out of 15 samples of OSCC, and membranous β-catenin expression was low or totally absent in 7 out of 15 samples of OSCC (data not shown). In various malignant tumors, reduced membranous β-catenin expression was associated with the emergence of invasion or metastasis [21-24]. Our results suggest that β-catenin function in cell-cell adhesion and translocation in the nucleus are related to proliferation and invasion of OSCC.

c-Myc and cyclin D1 have been identified as target genes of the Wnt/β-catenin pathway [14-17]. Tetsu and McCormick [25] reported that expression of cyclin D1 is strongly dependent on β-catenin/TCF and has a direct effect on cell proliferation in colon carcinoma cells. Furthermore, Kovesi and Szende [26] reported that expression of cyclin D1 increased during progression of the severity in oral leukoplakia. Our results showed elevated cyclin D1 expression in the epithelial dysplasia. In oral epithelium, cyclin D1 may participate in malignant transformation. c-Myc expression was elevated in dysplasia with nuclear expression of β-catenin than that without nuclear expression, although the difference in c-myc expression is not evident.

Our studies show the aberrant Wnt signaling pathway in the oral epithelial dysplasia. Wnt3, a typical member of the Wnt family that activates β-catenin mediated signaling [18,19], was not expressed in the normal oral epithelium. In contrast, Wnt3 expression was observed in the examples showing nuclear expression of β-catenin. Both the nuclear expression of β-catenin and Wnt3 expression were observed in oral leukoplakia with dysplasia, and therefore the aberrant Wnt signaling pathway may promote malignant transformation by triggering cyclin D1 expression and consequently uncontrolled progression into the cell cycle.

These lines of evidence suggest that nuclear accumulation of β-catenin plays an important role during malignant transition of oral leukoplakia through dysplasia. In addition to pathological examination of hematoxylin and eosin-stained sections for oral leukoplakia, immunochemical staining for β-catenin is useful for diagnosis of epithelial dysplasia and precancerous changes. Evaluation of other constituents for Wnt signaling, including GSK-3, CDK, Frizzled, and LRP5/6, helps us for determination and prognosis of precancerous change of dysplasia.

## Conclusion

As for the transcriptional activation of a target gene by β-catenin, elevated expression was detected in a squamous cell carcinoma and epithelial dysplasia of oral leukoplakia. Thus, Wnt/β-catenin pathway is considered to be involved in the progression of dysplasia in oral leukoplakia, as shown by nuclear expression of β-catenin and other components, including Wnt3 and cyclin D1.

## Methods

### Tissue samples

Formalin-fixed, paraffin-embedded samples of normal oral mucosa (6 examples), of oral squamous cell carcinoma (15 examples), and of oral leukoplakia (29 examples) were used. A fresh tissue of the oral cavity is also obtained at the time of biopsy or at oral surgery during treatment of cancerous disease after obtaining informed consent to the patients, and fixed in formalin before paraffin embedding. A histopathological diagnosis of each sample was performed by pathological staffs in the Department of Pathology in Kawasaki Medical School Hospital. According to the WHO diagnostic criteria for histological typing of cancer and pre-cancer of the oral mucosa [1], 12 examples from oral leukoplakia were diagnosed as epithelial dysplasia lesion (dysplasia). Dysplasia was diagnosed and graded as mild dysplasia (9 examples) and severe dysplasia (3 examples).

### Immunohistochemistry

Sections of 4 μm thickness in paraffin embedding tissue were treated with microwave for 10 minutes in 10 mM citrate buffer (pH 6) after de-paraffin processing, followed by washing with PBS twice for 10 minutes. To remove endogenous peroxidase activity, sections were treated in 3% hydrogen peroxide solution for 15 minutes, and subsequently in PBS twice for five minutes. The primary antibodies against β-catenin (Sigma-Aldrich), Wnt3 (Santa Cruz Biotechnology), cyclin D1 (Zymed Laboratories), and c-myc (Santa Cruz Biotechnology) were used at 1/400, 1/100, 1/50, and 1/50 dilution, respectively, in PBS by incubating at room temperature for 60 min. After washing with PBS, the sections were treated with biotinylated secondary antibody for 10 min and then with peroxidase-labeled streptavidin for 10 min (LSAB PLUS kit, DAKO, Japan), and the color was developed with 3,3'-diaminobenzidine tetrahydrochloride (LSAB PLUS kit, DAKO, Japan), followed by counter staining with Mayer's hematoxylin. As a negative control, primary antibodies were omitted and the sections were incubated with the secondary antibody alone.

### Evaluation of staining

For counting cells with nuclear and/or cytoplasm staining, three microscopic fields with 200-fold magnification were randomly chosen and the mean above 10% of the total cell numbers was deduced to be positive.

Evaluation of staining was carried out based on whether nuclear or cytoplasmic staining is detectable. Under microscopic field of 200-fold magnification, tissues are positive for β-catenin, if more than 10% of cells show cytoplasmic or nuclear staining. Randomly selected field in the tumor parenchymal region was used for determination of β-catenin positive cells in squamous cell carcinoma. Because signal intensity and distribution were different depending on the places in leukoplakia without dysplasia and in the normal oral epithelium, three fields were randomly selected to determine average ratio of a positive cell. In the Wnt3 staining, the cell membranous or cytoplasmic staining in the basal and spinous layer was regarded as positive. For cyclin D1 and c-myc, nuclear staining was regarded as positive. The cyclin D1 and c-myc staining was evaluated by counting at 200-fold magnification and calculating the percentage of positive cells. Statistical significance was estimated using Fisher's exact test, Mann-Whitney *U*-test, and Student's *t*-test.

## Abbreviations

GSK-3 – glycogen synthase kinase-3; LEF1 – lymphoid enhancer binding factor 1; LRP – low-density lipoprotein receptor-related protein; OSCC – oral squamous cell carcinoma; PBS – phosphate-buffered saline; TCF – T cell factor.

## Competing interests

The author(s) declare that they have no competing interests.

## Authors' contributions

KI and SI carried out immunohistochemical analysis and evaluation of staining, KI also drafted the manuscript, NW participated in evaluation of immunohistochemical staining and helped to draft the manuscript, HD and TH participated to collect specimens and performed in the statistical analysis, MH and TN participated in the design and coordination of the study, TN also conceived of the study and helped to draft the manuscript. All authors read and approved the final manuscript.
